# The existence of parenting styles in the owner-dog relationship

**DOI:** 10.1371/journal.pone.0193471

**Published:** 2018-02-23

**Authors:** Ineke R. van Herwijnen, Joanne A. M. van der Borg, Marc Naguib, Bonne Beerda

**Affiliations:** Department of Animal Sciences, Behavioural Ecology Group, Wageningen University and Research, Wageningen, The Netherlands; Universidade do Porto Instituto de Biologia Molecular e Celular, PORTUGAL

## Abstract

Parents interact with children following specific styles, known to influence child development. These styles represent variations in the dimensions of demandingness and responsiveness, resulting in authoritarian, authoritative, permissive or uninvolved parenting. Given the similarities in the parent to child and owner to dog relationships, we determined the extent to which parenting styles exist in the owner to dog relationship using the existing Parenting Styles and Dimensions Questionnaire for the parent-child relationship and an adapted version for dog owners. Items on the parenting of children/dogs were rated for applicability on a five-point Likert scale by 518 Dutch dog owning parents. Principal Component Analyses grouped parenting propensities into styles, with some marked differences between the findings for children and dogs. Dog-directed items grouped into an authoritarian-correction orientated style, incorporating variation in demandingness and focussing on correcting a dog for behaviour verbally/physically, and in two styles based on authoritative items. An authoritative-intrinsic value orientated style reflected variation in mainly responsiveness and oriented on the assumed needs and emotions of the animal. A second authoritative-item based style, captured variations in demandingness and responsiveness. We labelled this style authoritative-training orientated, as it orientated on manners in teaching a dog how to behave in social situations. Thus, we defined dog-directed parenting styles and constructed a Dog-Directed Parenting Styles and Dimensions Questionnaire along the lines of the existing theoretical framework on parenting styles. We did not find a dog-directed parenting style of being permissive or uninvolved, which we attribute to a study population of devoted dog owners and our findings should be interpreted with this specific study population in mind. We found evidence of dog-directed parenting styles and provide a fundament for determining their possible impact on the different aspects of a dog’s life.

## Introduction

Strategies of parents to raise their children are recognized as parenting styles, reflecting relatively stable patterns in parenting behaviour and goals the caretaker has with parenting the child. Parenting styles are relevant because of their effects on the development and well-being of children. They could exist also in the owner-dog relationship and, possibly, present a pathway to improve dog behaviour and welfare. The dimensions underlying four main parenting styles [1, 2, 3] are demandingness and responsiveness. Demandingness refers to the monitoring of the child and practicing of confrontive control. Monitoring provides structure, order and predictability, where confrontive control ‘teaches a child to behave well’ by discouraging disruptive behaviour and enforcing rules in a goal-orientated and reasonable way. Responsiveness represents emotional warmth and supportive actions, reflecting the degree to which a parent responds to the child’s needs and wishes. Thus, where demandingness places demands on the child and directs it, responsiveness allows the child to be seen and heard. Demandingness and responsiveness are separate dimensions, not contrasting elements, and it is assumed that optimal parenting is characterized by scoring highly on both dimensions [4]. Up to seven different parenting styles have been defined [3, 4], but here we focus on the three original ones, being authoritarian, authoritative and permissive (indulgent), plus the uninvolved style [4]. The latter is known as disengaged or neglectful and this style was added to the original three [5]. The authoritarian style manifests as being demanding, exerting high levels of control, with low levels of responsiveness [3]. Children are expected to follow the strict rules set by parents, reasoning is not explained and failure to adhere to rules results in punishment. The authoritative style combines strong tendencies in both dimensions of demandingness and responsiveness [3]. High demands are placed on children, which are expected to behave properly, but judgements, values and goals are explained to them and parents are more willing to negotiate. The permissive style involves low levels of demandingness, but strong responsiveness [3]. Children have few rules to follow and little is demanded of them. Lastly, the uninvolved style scores low on both demandingness and responsiveness, resulting in ‘least effort parenting’. Few demands are made of the children and communication is minimal on rules as well as on the child’s needs and emotions [3].

To determine the effects of the parenting styles on children, in human psychology the Parenting Styles and Dimensions Questionnaire (PSDQ) is used. The PSDQ was specifically developed to identify parenting practices based on self-reports by parents of (pre)school-aged children [6]. The original version, also known as the Parenting Practices Questionnaire (PPQ), consisted of 62 items (62-PSDQ) and was later shortened to 32 items (32-PSDQ) [6, 7]. Both measure on the authoritarian, authoritative and permissive parenting style. For the permissive parenting style, which was suggested to measure inconsistency in parenting more than permissiveness, reliability and validity may be limited when using the 32-PSDQ [8]. The uninvolved parenting style was measured less often in studies, and thesis work by Blakely Kimble [9] defined this style indirectly with existing PSDQ items for the other three parenting styles. She validated measures of uninvolved parenting against parenting practices, maternal depression and interactions with the child at meal times. Factor analysis of a questionnaire filled out by 378 mothers of first grade children revealed associations for uninvolved parenting with items on the use of threats, lack of following through, rejection and lack of discipline and, inversely, with regulation and reasoning. Such aspects of low control and high rejection as characteristics of uninvolved parenting correspond with earlier reports of minimal parenting effort/time [5] and of lax behavioural control as well as rejection [4].

Parenting styles influence child development, with the authoritative style being optimal. Academic competence and self-reliance for instance, as measured on a three-point Likert-scale in 4,081 fourteen- to eighteen-year-old US children, was significantly higher in authoritatively parented children than in children parented otherwise [10]. Misconduct scored lower concomitantly, and self-reliance was significantly higher for authoritatively guided children compared to those parented with an authoritarian or uninvolved style [10]. Having one or two authoritative parents protected against delinquency and depression in eight-grade adolescents from 451 US families [11] and authoritative parenting promoted self-esteem, subjective well-being, secondary education results and continuing education in 1,456 British fifteen-year-olds [12]. Clearly, the style in which children are parented directs their development and well-being, raising the question if the same applies to companion animals like dogs.

Research on the identification of parenting styles and the effects of parenting is abundant in human psychology, but almost non-existent in companion animal sciences [13]. Consequently, we may miss out on opportunities to guide the behavioural development of dogs and improve their well-being. Parenting could play an important role in human-dog interactions as, intraspecific, mothering and nursing style influence later behaviour of guide dogs [14] and many people view and treat their dogs similar to children. Almost half of the dog owners (48%) regarded their dog as a child or close companion where the other half (52%) indicated the dog to be ‘part of the family’, in a US-survey on 343 adopters of cats and dogs [15]. Regarding the dog as a family member was indicated by 93% of 14,004 dog owners in Germany [16]. The option ‘child’ was not offered as choice, and options such as ‘hobby’ (50%) were indicated less frequently than ‘family member’ [16]. It remains somewhat speculative what the dog as a family member encompasses precisely, but in 711 dog owners, of which 98% regarded their dog a family member, over 40% celebrated their dog’s birthday and shared snacks with them frequently [17]. Behaviour of dog owners towards their dogs provides further insights in the nature of the owner-dog bond. The way dog owners talk, show affiliative behaviour and play after separation, led to the suggestion that modern dog ownership can be typed as interspecific parental behaviour [18]. Dogs seem to exploit our tendencies towards empathy, nurturing and anthropomorphism, and tap into mechanisms that underlie parent-child relationships [19]. This view has been specified by the idea that dogs tap into the oxytocin loop that plays a role in mother-child attachment, based on differential urinary oxytocin levels in dog owners who experienced different durations of intentional eye contact with their dog [20]. The oxytocin loop is important in both attachment and in (eliciting) caregiving behaviour in infant-parent relationships and if dogs are indeed able to activate oxytocin based mechanisms of bonding, this provides additional argumentation for owner behaviour directed at dogs to resemble that of parenting behaviour.

Sufficient argumentation exists to assume a correspondence in behaviour patterns that parents show towards children and dogs, but there is need for further scientific evidence. Here we determined styles in the parenting of dogs by using an (adapted) PSDQ, as the existence of dog-directed parenting styles could bring new ways to improve the owner-dog relationship and latter’s quality of life. We validate the psychological construct of parenting styles in the owner-dog relationship and determine if parenting styles express similarly or dissimilarly in the relationships of owner to dog as in parent to child.

## Methods

### Web-based survey

Dog owning parents of at least one child (age not reported) participated in a Dutch language web-based survey, after having been recruited via pet stores, vets, dog schools, human schools, (animal) foodbank organisations, online and hardcopy magazines on (human) parenting as well as on (companion) animals. The online survey’s introduction explained the purpose of the research and the study did not involve treatments or interventions in the life of respondents or their dogs. The questionnaire was not repeated, meaning it did not interfere significantly with normal daily life, and did not include questions that were psychologically burdening. This exempts the study from review by our ethics committee, according to the guidelines of Wageningen University Medical Ethics Review Committee (Medisch Ethische Toetsingscommissie van Wageningen University, METC-WU). Informed consent was not obtained as respondents choice to participate freely via internet and the purpose of the research was stated at the start of the online survey.

The survey ran from May 2016 till November 2016 and included three main parts, with the first consisting of 21 questions (items) on the background of the owner, her/his household and the dog. We did not collect privacy sensitive information for the purpose of our research, such as names and addresses of respondents. The second part contained 62 items on dog-directed parenting, explained below. The third held 62 items on child-directed parenting, being the Parenting Styles and Dimensions Questionnaire (62-PSDQ) developed by Robinson et al. [6, 7] for measuring child-directed authoritarian, authoritative and permissive parenting styles along dimensions of demandingness and responsiveness. This 62-item questionnaire was analysed also as the shorter 32-PSDQ. In the 62-PSDQ, 20 items measure the authoritarian style, 27 items the authoritative and fifteen the permissive. In the 32-PSDQ, twelve items measure the authoritarian style, fifteen the authoritative and five the permissive. We calculated the uninvolved style following Blakely Kimble [9] as well as following Baumrind’s [4] ideas on it representing weak behavioural control and strong rejection (for the items see S1 Appendix).

We wanted to validate the child-directed PSDQ for measuring dog-directed parenting and adapted existing items to situations dog owners encounter when raising their dog. For example, the original item ‘I spank when my child is disobedient’, was reformulated to ‘I use a corrective slap when my dog misbehaves’ (S1 Table lists all items).

Both sets of items on parenting directed at children and dogs were translated into Dutch and pretested with five Dutch native speakers (male and female, aged 39–53 and responsible for the care of both dog and child) to detect possible obscurities in the questions. PSDQ items were measured on a five-point Likert scale, rating the likelihood of scenarios occurring as never (score 0), nearly never (1), neutral (defined as about half of the time, 2), nearly always (3) and always (4). Parenting style scores were calculated following Robinson et al. [6] by summing scores for items on a same parenting style, with some items being scaled reversely, and expressing the sums as percentages of the theoretical maximum.

### Statistical analyses

Statistical analyses were performed with GenStat (18th edition). Item scores were analysed for associations by standard Principal Component Analyses (PCA, [21]) based on correlation matrices and with the number of principal components set at five. Principal components are uncorrelated and orthogonal, expressing in patterns of eigenvectors as representations of direction. Scaling is represented by eigenvalues, which we integrated by calculating loadings as eigenvectors multiplied by the square root of eigenvalues. Principal components were not rotated and we regarded loadings ≥ │0.4│ as meaningful, indicating that an item fitted into a component, or candidate dimension of parenting. The meaningfulness of a component is indicated by the amount of variance in the data set that it explains. Construct validity was assumed if meaningful components grouped items logically according to the existing framework on child-directed parenting styles. Correlations between scores for parenting styles directed at the child and those directed at the dog were studied both with Pearson’s and Spearman’s (r_s_) rank, resulting in similar outcomes and only the latter are presented. Descriptive results are presented as means ± standard deviations (s.d.), with median values and lower/upper quartile indicated as additional information on parenting style distribution.

We constructed a short Dog-Directed Parenting Styles and Dimensions Questionnaire (DD-PSDQ) based on the statistical outcomes of the first PCA on the dog-directed 32-PSDQ. We dropped four items from this first PCA, retained the other twenty items with loadings ≥ │0.4│. Dropped items associated with a different parenting style than in Robinson’s original PSDQ. On the kept items we ran a second PCA and added two items from the 62-PSDQ to create a more balanced set of scales. The two chosen items had the highest loadings of the items additional to the 32-PSDQ. Internal consistency of the newly constructed DD-PSDQ dimensions was tested with Cronbach’s alpha.

## Results

### Participants and their dogs

Dog owning parents of at least one child (age not specified) participated in the study. The number of respondents was 518, but with the occasional items remaining blank (missing values). In the following, percentages are given relative to this total and the precise sample sizes are indicated. The majority of the respondents was female (91%, *N =* 470; male: 8%, *N* = 43) and more than three quarters (83%, *N* = 428) had completed upper secondary education or higher. Age of the respondents was indicated in seven age categories and most belonged to age groups 35–44 years (32%, *N =* 164) and 45–54 years (35%, *N =* 183). The mean (±s.d.) number of children was 1.7±0.8 on a four-point scale of one to four or more. Most respondents had one child (43%, *N =* 205) or two (44%, *N =* 213), 9% (*N =* 45) had three children and 4% (*N =* 19) had four or more.

The mean (±s.d.) number of dogs held by respondents was 1.6±0.9, again on a four-point scale of one to four or more. More than half of the respondents had one dog (58%, *N =* 302), 28% (*N =* 144) had two, 9% (*N =* 44) had three and 5% (*N =* 27) had four or more dogs. Dogs were of varying breeds and the vast majority of dogs had been purchased in part for companionship (92%, *N =* 475) and/or walking (66%, *N =* 341), 24% (*N =* 126) had been bought, also, for partaking in dog sports, 13% (*N =* 67) for the owner to feel safer, 7% (*N =* 38) for breeding, 7% (*N =* 37) for work, 6% (*N =* 30) for dog shows, 4% (*N =* 23) for animal assisted therapy and 5% (*N =* 25) for guarding and/or as resident (‘yard-kept’) dog, meaning the dog resides mainly at the premises, not indoors. Nearly three quarters of the respondents’ dogs were reported to be always inside the house when the owner was inside (71%, *N =* 369), for 23% (*N =* 121) this was ‘mostly’ and for 4% (*N* = 16) this was ‘nearly never’ or ‘never’.

### Parenting style scores by conventional methods

Credibility of the 32-item Parenting Styles and Dimensions Questionnaire (PSDQ) for measuring the three basic parenting styles was confirmed by the analyses of scores for both child- and dog-directed parenting styles. The measures of 32-PSDQ and 62-PSDQ correlated significantly for both child-directed parenting (authoritarian r_s_ = 0.90, P<0.001, authoritative r_s_ = 0.95, P<0.001, permissive r_s_ = 0.79, P<0.001; *N* = 518 for all comparisons) and dog-directed parenting (authoritarian r_s_ = 0.94, P<0.001, authoritative r_s_ = 0.95, P<0.001, permissive r_s_ = 0.71, P<0.001; *N* = 518 for all comparisons; see Table 1 for the descriptive mean scores of child- and dog-directed parenting). For uninvolved parenting, scores between the Blakely Kimble and Baumrind method correlated again significantly, but at lower levels than the conventionally measured parenting styles (child-directed parenting r_s_ = 0.62, P<0.001, dog-directed parenting r_s_ = 0.35, P<0.001; *N* = 518 for all comparisons).

**Table 1:** Descriptive mean scores of child- and dog-directed parenting. Dog owning parents (*N* = 518) reported on the parenting of their children by answering 62 items of the Parenting Styles and Dimensions Questionnaire (PSDQ) and on their dogs by answering 62 adapted items, both on a five-point Likert scale. Parenting style scores were calculated following standard procedures from both the full 62-item PSDQ and the shortened 32-item version, and expressed as percentage of the theoretical maximum. Presented are the mean child- and dog-directed parenting scores ± s.d. (range), as well as the medians and the threshold values at the lower and upper quartile that demarcate the range of 50% middle values.

**Parenting style**				
	Dog-32	Child-32	Dog-62	Child-62
Authoritarian	23.2±13.6	14.9±12.3	27.5±12.3	20.7±10.8
	(0–79.2)	(0–79.2)	(2.5–77.5)	(0–78.8)
	20.8	12.5	26.3	18.8
	(12.5–31.3)	(6.3–20.8)	(18.8–35.0)	(13.2–26.3)
Authoritative	70.5±13.0	83.4±12.7	72.4±12.0	83.3±11.9
	(31.7–100)	(0–100)	(27.9–98.1)	(0–100)
	71.7	85.0	73.2	85.2
	(61.7–80.0)	(76.7–93.3)	(64.8–80.6)	(76.9–91.7)
Permissive	23.3±13.7	31.5±16.6	28.2±10.2	28.7±10.6
	(0–75.0)	(0–100)	(3.3–68.3)	(1.7–71.7)
	20.0	30.0	26.7	28.3
	(15.0–30.0)	(20.0–40.0)	(21.7–33.3)	(21.7–35.0)
Uninvolved-Blakely			24.2±9.6	24.2±11.5
			(6.3–54.2)	(0–72.7)
			22.9	25.0
			(16.7–29.2)	(15.9–31.8)
Uninvolved-Baumrind			30.6±6.9	21.6±7.1
			(10.7–56.3)	(4.8–52.4)
			29.8	21.4
			(25.0–35.7)	(16.7–25.0)

Dog-directed parenting and child-directed parenting styles correlated significantly (32-PSDQ: authoritarian r_s_ = 0.59, P<0.001, authoritative r_s_ = 0.46, P<0.001, permissive r_s_ = 0.44, P<0.001; 62-PSDQ: authoritarian r_s_ = 0.55, P<0.001, authoritative r_s_ = 0.50, P<0.001, permissive r_s_ = 0.48, P<0.001; Blakely Kimble uninvolved r_s_ = 0.49, P<0.001, Baumrind uninvolved r_s_ = 0.31, P<0.001; *N* = 518 for all comparisons). For the three basic styles, the correspondence between parenting children and dogs explained in the range of 19–35% of the variation and for the uninvolved style 10–24% of the variation was explained.

### Associations between items on parenting styles

Dimensions indicative of parenting styles were found in the Principal Component Analyses (PCA) with sets of 32 and 62 items on the everyday ways in which dog owning parents (*N* = 518) parented their children and dogs (mean item scores for dog-directed parenting are available in S1 Table).

On the parenting of children, the items associated as expected (32-PSDQ: authoritative component explaining 23.0% of variation, authoritarian: 15.4%, permissive: 6.0%; for details on loadings see S2 Table), but a component in correspondence with an uninvolved style was not detected.

For dog-directed parenting, components were found reflecting authoritarian and authoritative parenting (32-PSDQ: authoritarian: 16.3%, authoritative: 10.7% and 8.0%, for details see Table 2; 62-PSDQ: authoritarian: 8.6%, authoritative: 12.8% and 7.3%, for details on loadings see S3 Table). The authoritarian component captured items on verbal/physical forcefulness and corrections for unwanted behaviour. Authoritative parenting emerged as two different components in dogs, indicating that authoritative parenting directed at dogs differentiates from human child directed parenting. The first component with items from the original authoritative style explained 10.7% of the variation and captured items of orientation on animal intrinsic value and animal emotions, with respondents varying in taking a dog’s needs and emotions as a starting point for parenting practices. Included were items like ‘I allow my dog to give input on decisions for instance with regard to the route we follow on walks’ and ‘I give comfort when my dog is upset’. The second component with items on acting authoritative explained 8.0% of the variation and captured items of orientation on training as a starting point for parenting practices. It held items such as ‘I use more or higher value reward (food or toy) when I believe my dog should really do something in a situation’ and ‘I practice behaviour step by step with my dog, so I am sure he/she understands what I ask of him/her’. The PSDQ items for assessing dog-directed parenting thus grouped as expected into an authoritarian parenting style, but separated into two different styles where it regarded authoritative parenting. One of the latter two styles included items of permissive parenting, but the PCA did not identify a distinct permissive dog-directed parenting style, nor an uninvolved style.

**Table 2:** Dog-directed parenting components. Dog owning parents (*N* = 518) reported on dog-directed parenting in 32 items adapted from the Parenting Styles and Dimensions Questionnaire (PSDQ). Answers on a five-point Likert scale were analysed by Principal Component Analysis and presented are the loadings ≥ |0.4| and percentages of variation explained by the main components, which represented dimensions of parenting authoritarian, authoritatively-intrinsic value orientated and authoritatively-training orientated.

**Item**	**Variation explained**		
	(latent root)		
	16%	11%	8%
	(5.2)	(3.4)	(2.6)
	Authoritarian	Authoritative-intrinsic value orientated	Authoritative-training orientated
I use a corrective slap when my dog misbehaves.^AN^	-0.67		
I raise my voice to make my dog improve.^AN^	-0.67		
I yell or shout when my dog misbehaves.^AN^	-0.65		
I use physical punishment (for instance a slap or a correction chain) as a way to improve my dog’s behaviour.^AN^	-0.64		
I can explode in anger towards my dog when he does something he knows I don’t want him to do.^AN^	-0.63		
I grab my dog when he is being disobedient.^AN^	-0.62		
I use a poke of my finger, or short kick to snap my dog out of it when it misbehaves.^AN^	-0.60		
I scold or criticize when my dog's behaviour doesn't meet my expectations.^AN^	-0.58		
I use threats as punishment without feeling need for justification towards my dog.^AN^	-0.51		
When I ask my dog to do something, he should do so, because I said so and I am its boss.^AN^*	-0.47		
I threaten with punishments towards my dog and do not actually do them.^PM^^!^	-0.42		
I allow my dog to give input on decisions for instance with regard to the route we follow on walks.^AV^		0.68	
I give comfort when my dog is upset.^AV^		0.64	
I spoil my dog.^PM^^!^		0.57	
I take my dog's desires into account before asking him to do something.^AV^		0.57	
I am responsive to my dog's feelings or needs.^AV^		0.52	
When I ask my dog to do something, he should do so, because I said so and I am its boss. ^AN^^!^*		-0.50	
I encourage my dog to show how it feels, it is allowed to growl for instance, when uncomfortable.^AV^		0.50	
I give into my dog when he causes a commotion about something or doesn’t do something I want it to.^PM^^!^		0.48	
I take into account my dog's preferences in making plans.^AV^		0.47	
I use more or higher value reward (food or toy) when I believe my dog should really do something in a situation.^AV^			0.66
I practice behaviour step by step with my dog, so I am sure he understands what I ask of him.^AV^			0.60
I think about why rules should be obeyed by my dog.^AV^			0.58
I give praise when my dog is good.^AV^			0.57

^AN^—Authoritarian item in the original PSDQ.

^AV^—Authoritative item.

^PM^–Permissive item.

^!^—Item scoring in a different PSDQ dimension than found originally by Robinson et al. [6].

*—Item surfacing in two PCA-components.

Outcomes from the PCA on 62 dog-directed PSDQ items were in line with the findings based on 32 items, giving us further confidence that the shorter 32-questionnaire is a sound alternative for assessing possible dog-directed parenting styles, and next, we constructed a specific Dog-Directed Parenting Styles and Dimensions Questionnaire.

### Dog-Directed Parenting Styles and Dimensions Questionnaire

To construct a Dog-Directed Parenting Styles and Dimensions Questionnaire (DD-PSDQ) we run a second PCA on the dog-directed 32-PSDQ items shown in Table 2, excluding the four items loading on a different parenting style than in Robinson’s original PSDQ. This resulted in four main components that included eighteen items loading ≥ │0.4│ (S4 Table). We interpreted the four PCA components as parenting styles that were authoritarian-verbal correction oriented (22% of the variation explained, four items), authoritarian-physical correction oriented (6%, four items), authoritative-intrinsic value orientated (12%, six items) and authoritative-training orientated (8%, four items). We labelled the latter two styles ‘authoritative’ to indicate that all items in these two styles come from this original parenting style, as defined in earlier research on child-directed parenting. Authoritativeness in dog-directed parenting was found to be distinct from child-directed parenting as it divided in two separate components.

Next, to create a more balanced number of items across three main dimensions, we added the two authoritative-training orientated items from the PCA on the 62-PSDQ that had the highest loadings of the items additional to the 32-PSDQ. These being ‘I practice certain behaviour with my dog before asking this behaviour in a more difficult situation’ (loading: 0.70) and ‘I channel my dog's misbehaviour into a more acceptable activity’ (0.60). So, within the assumed dog-directed style of authoritarian parenting, the use of voice and physical contact varied independently, but for reasons of compatibility with the existing theoretical framework we decided to merge the two independent components into one authoritarian-correction oriented style. To us, these two styles do not reflect differences in the dimension of demandingness, but merely in the way of expressing it verbally or physically. Thus, our end DD-PSDQ consisted of 20 items, eight measuring the authoritarian-correction orientated style, six the authoritative-intrinsic value orientated style and six the authoritative-training orientated style (Table 3). Tests for internal consistency confirmed that the items within each of the three DD-PSDQ dimensions measured the same construct. Cronbach’s alphas were 0.80 for the authoritarian-correction orientated style, 0.74 for the authoritative-intrinsic value orientated and 0.77 for the authoritative-training orientated style. The DD-PSDQ parenting styles scored on average 22.5±16.2% (ranging from 0–93.8) for authoritarian-correction orientated, 59.6±19.3% (0–100) for authoritative-intrinsic value orientated and 79.4±16.3% (8.3–100) for authoritative-training orientated. Scores for the newly constructed DD-PSDQ styles correlated significantly with those of the 32-PSDQ on dog-directed parenting, with r_s_ = 0.94 (P<0.001, *N* = 518) for 32-PSDQ authoritarian and DD-PSDQ correction orientated, r_s_ = 0.88 (P<0.001) for 32-PSDQ authoritative and DD-PSDQ intrinsic value orientated, and r_s_ = 0.65 (P<0.001) for 32-PSDQ authoritative and DD-PSDQ training orientated. These relatively high correlations indicate that measures by the DD-PSDQ connect to the original measuring tool (with consistencies as well as marked differences) and, likely, the underlying theoretical framework.

**Table 3:** Dog-directed Parenting Styles and Dimensions Questionnaire. The Dog-Directed Parenting Styles and Dimensions Questionnaire (DD-PSDQ) as constructed from the adapted 32-PSDQ with the addition of two elements from the 62-PSDQ to create a more balanced set of scales.

***Authoritarian–correction orientated***
I yell or shout when my dog misbehaves
I scold or criticize when my dog's behaviour doesn't meet my expectations
I can explode in anger towards my dog when he does something he knows I don’t want him to do
I raise my voice to make my dog improve
I use physical punishment (for instance a slap or a correction chain) as a way to improve my dog’s behaviour
I use a corrective slap when my dog misbehaves
I use a poke of my finger, or short kick to snap my dog out of it when it misbehaves
I grab my dog when he/she is being disobedient
***Authoritative–intrinsic value orientated***
I allow my dog to give input on decisions for instance with regard to the route we follow on walks
I take my dog's desires into account before asking him to do something
I am responsive to my dog's feelings or needs
I encourage my dog to show how it feels, it is allowed to growl for instance, when uncomfortable
I give comfort when my dog is upset
I take into account my dog's preferences in making plans
***Authoritative–training orientated***
I give praise when my dog is good
I practice behaviour step by step with my dog, so I am sure he understands what I ask of him
I use more or higher value reward (food or toy) when I believe my dog should really do something in a situation
I think about why rules should be obeyed by my dog
I practice certain behaviour with my dog before asking this behaviour in a more difficult situation
I channel my dog's misbehaviour into a more acceptable activity

## Discussion

Here we show how the concept of child-directed parenting styles applies to dog-directed parenting, with distinct differences like the separation into two authoritative styles. We found an expected style of authoritarian dog-directed parenting, but likely our study population of devoted dog owners prevented us from detecting styles of permissive or uninvolved parenting. Adapting an existing child-directed PSDQ for use with dogs without adding any new items facilitates that expected parenting styles resurface in the data, which makes our finding of differences in parenting dimensions directed towards children and dogs especially salient.

We confirm that the 32-item Parenting Styles and Dimensions Questionnaire (32-PSDQ) is a valid alternative to its lengthier counterpart of 62 items and demonstrate how it can be re-constructed into a 20-item Dog-Directed Parenting Styles and Dimensions Questionnaire (DD-PSDQ) that shows good internal consistency on its three scales and associates logically with the original 32-PSDQ dimensions of authoritarian and authoritative parenting.

Our finding of dog-directed parenting styles, at least for authoritarian and authoritative parenting, expands on earlier research indicating that dog owners typically experience strong and family-like bonds with their dogs [16, 17]. Similarities between child-directed and dog-directed parenting styles as documented here, indicate consistency of parenting styles. This consistency was shown before over time [22, 23] but not across species, giving rise to a plea for research on interspecies parenting styles [13]. Consistency of parenting styles over time supports the existence of long-term parental attitudes, objectives and patterns of practices, which makes them different from short-term parenting behaviours [24]. The latter may vary over time, for example as requirements change for the maturing child [3]. We found proof of parenting styles applying to the interspecies owner-dog relationship and detected distinct patterns of dog-directed parenting that are authoritarian-correction orientated and authoritative-intrinsic value/training orientated, resembling parenting styles directed at children without being identical to them. The dissimilarities possibly result from varying orientations that humans have towards dog ownership. Child-directed parenting styles are known to reflect underlying orientations, values and goals of the parent [4] and in dog owners, an intrinsic orientation was distinguished from an extrinsic orientation in a small qualitative study of seven thorough in-home interviews. The intrinsically orientated owners viewed their dog as an individual, whereas the extrinsically orientated owners had the dog with the purpose to build their personal identity through exerting control over the dog and/or gaining status from it [25]. Intrinsic type of orientations have been categorized as protectionistic and humanistic in a qualitative study on 28 dog owners [26], with the extrinsic type resembling a dominionistic orientation. Protectionistic owners view dogs predominantly as animals with their own interests, humanistic owners adopt an anthropomorphic stance and dominionistic owners value animals especially for their uses [26]. The presently found authoritarian-correction orientated style of dog-directed parenting could be driven by a more extrinsic coercive orientation towards dogs, and the two authoritative styles could possibly fit an intrinsic orientation. Further research should clarify how animal orientations and dog-directed parenting styles may be related.

The two dog-directed parenting styles characterized by low demandingness, i.e. permissiveness and uninvolvedness, remained undetected in our analysis. It is important to realise that this does not exclude their existence. We suspect our study population to have held only few dog owners with a permissive or uninvolved parenting style as completing the lengthy survey, which was necessary to address the different aspects for developing a DD-PSDQ, took some effort and commitment. The permissive and uninvolved style are known for making relatively little effort in parenting [27] and people who minimize efforts in raising their children/dogs are unlikely to fill out an extensive time-consuming questionnaire on the subject of parenting. Alternatively, variation along the dimension of demandingness may be less pronounced in the owner-dog relationship than in the parent-child relationship and the authoritative-intrinsic value orientated parenting style could be the dog-directed variant of permissiveness. Our study does not confirm or rule out dog-directed parenting styles that are permissive or uninvolved as the study population was too particular by the participants’ assumed strong commitment to their dogs and having at least one child. An uninvolved style of parenting may have remained unnoticed by us and requires further attention especially as neglect of children is known to associate with that of animals [28, 29, 30]. Our findings unlikely apply to the whole population of Dutch dog owners and further research is necessary to strengthen and clarify the concept of dog-directed parenting styles.

In our measurement of the parenting styles, we deployed the 32-PSDQ and 62-PSDQ and confirmed the shorter 32-PSDQ as a valid tool amongst the several parenting style measurements that have been used in (human directed) research [8]. The 62-PSDQ was developed originally for the purpose of determining parenting styles through self-report by parents of (pre)school-aged children. It resulted from a study on 534 fathers and 717 mothers answering a 133-item questionnaire. Successive factor analyses organized the 62-items with good internal consistency [6] and the shorter, 32-item version of the PSDQ was produced later on [7]. Reliability and validity has been addressed mainly for the 32-PSDQ [7], revealing that the permissive style, which is measured by the least number of items, scores lowest on reliability [8]. We noticed this too and scores for (child- and dog-directed) permissive parenting showed lower, but still significant, correlations between 32- and 62-PSDQ, than the authoritarian and authoritative styles.

We constructed a 20-item DD-PSDQ that is compact, requires little effort to complete and captures variation in an authoritarian-correction orientated style and in two authoritative styles, with a connection proven to the original 32-PSDQ measuring tool and theoretical framework. For capturing the full spectrum of four parenting styles the 32-PSDQ may yet prove to be the better tool though. As explained, the DD-PSDQ does not capture distinct styles of parenting permissively or uninvolved. Further research with a broad spectrum of dog owners, including those who are less inclined to partake in studies, is needed to determine the existence of permissive and uninvolved styles in dog-directed parenting. Until this is resolved, we suggest to base research on dog-directed parenting on both the 32-PSDQ and the DD-PSDQ. The 32-PSDQ has the potential to capture all four parenting styles, should the styles of permissiveness and uninvolvedness prove to be relevant in dog-directed parenting. The DD-PSDQ is useful especially in measuring variation in dog-directed parenting among common dog owners who vary in responsiveness and to at least some degree in demandingness, though extremes of low demandingness may be missed.

Being able to assess dog-directed parenting styles is a first step to promote appropriate parenting in dogs. In humans, the authoritative parenting style is known to give optimal child outcomes, in terms of high school/academic performance [10, 12] and high self-reliance/esteem levels [11, 12]. Possibly in dogs also, positive effects on behaviour and welfare can be achieved through authoritative dog-directed parenting. Dog owners often find themselves presented with unwanted behaviour such as aggression and this may challenge a close owner-dog relationship [31, 32, 33]. New ways of preventing such disruptions of the relationship between owner and dog, may be found in steering dog owners towards desired parenting styles. Research on farm animals has already shown beneficial effects for animal welfare of targeting underlying elements of human-animal interactions. Stock handler-animal interactions for instance, improved after cognitive-behavioural intervention procedures with positive effects on the welfare of pigs and cows [34, 35, 36]. To date, similar studies on improving the human-dog relationship seem lacking. Promoting appropriate parenting of dogs, possibly by targeting underlying elements of the owner-dog relationship, such as animal orientations, may offer opportunities to improve canine quality of life following what is known about child-directed parenting.

## Supporting information

S1 Appendix Items measuring the uninvolved parenting style. PSDQ items in the uninvolved style following Blakely Kimble (2009) and Baumrind (2013)
(PDF)

S1 Table Overview of PCA-item loadings. Results of loadings after rotation from PCA-analysis for 32/62-PSDQ (Robinson et al., 1995) and new dog-directed parenting styles in 518 Dutch dog owning parents; presented are the loadings ≥ |0.4|.
(PDF)

S2 Table 32-item child-directed PSDQ Principal Component Analysis. Dutch dog owning parents (N = 518) reported on child-directed parenting in 32 items adapted from the Parenting Styles and Dimensions Questionnaire (PSDQ). Answers on a five-point Likert scale were analysed by Principal Component Analysis and presented are the loadings ≥ |0.4| and percentages of variation explained by the main components, which represented dimensions of parenting authoritatively, authoritarian and permissively.
(PDF)

S3 Table 62-item dog-directed PSDQ Principal Component Analysis. Dutch dog owning parents (N = 518) reported on dog-directed parenting in 62 items adapted from the Parenting Styles and Dimensions Questionnaire (PSDQ). Answers on a five-point Likert scale were analysed by Principal Component Analysis and presented are the loadings ≥ |0.4| and percentages of variation explained by the main components, which represented dimensions of parenting authoritatively and authoritarian.
(PDF)

S4 Table Second step 32-item dog-directed PSDQ Principal Component Analysis. Dutch dog owners (N = 518) filled out a 32-item Parenting Styles and Dimensions Questionnaire (PSDQ) adapted for assessing dog-directed parenting styles. Answers on a five-point Likert scale were analysed by Principal Components Analysis, omitting items with loadings < │0.4│for the main components. Presented are the final outcomes on eighteen items with loadings ≥ |0.4| and percentages of variation explained by two components of parenting authoritarian-correction orientated (two times four items), one component of authoritative-intrinsic value orientated (six items) and one of authoritative-training orientated (four items), together explaining 48% of variation.
(PDF)

## References

[1] Barber BK. Parental psychological control: revisiting a neglected construct. Child Dev. 1996; 67(6):3296–3319. 9071782

[2] Baumrind D. The influence of parenting style on adolescent competence and substance use. J Early Adolesc. 1991;11(1):56–95.

[3] Baumrind D, Lazelere RE, Owens EB. Effects of preschool parents’ power assertive patterns and practices on adolescent development. Parent Sci Pract. 2010;10:157–201.

[4] Baumrind D. Authoritative parenting revisited: history and current status In: Larzelere RE, Sheffield Morris A, Harrist AW, editors. Authoritative Parenting. Synthesizing Nurturance and Discipline for Optimal Child Development. Washington: American Psychological Association; 2013 pp.11–34.

[5] Maccoby EE, Martin JA. Socialization in the context of the family: Parent-child interaction In: Mussen PH, Hetherington EM, editors Handbook of child psychology: Vol.4, Socialization, personality, and social development. 4th ed. New York: Wiley; 1983 pp.1006–1017.

[6] Robinson CC, Mandleco B, Olsen SF, Hart CH. Authoritative, authoritarian and permissive parenting practices: development of a new measure. Psychol Rep. 1995;77:819–830.

[7] Robinson CC, Mandleco B, Olsen SF, Hart CH. The parenting styles and dimensions questionnaire In: Perlmutter BF, Touliatos J, Holden GW, editors. Handbook of family measurement techniques. Vol.3 Instruments & index. Sage: Thousand Oaks; 2001 pp.319–321.

[8] Olivari MG, Tagliabue S, Confalonieri E. Parenting Style and Dimensions Questionnaire: a review of reliability and validity. Marriage Fam Rev. 2013;49:465–490.

[9] Blakely Kimble AB. The Parenting Styles and Dimensions Questionnaire: a reconceptualization and validation. Thesis approved by Hubbs-Tait L, submitted to the Faculty of the Graduate College of the Oklahoma State University in July 2014. Stillwater: Oklahoma State University; 2009.

[10] Lamborn SD, Mounts NS, Steinberg L, Dornbusch SM. Patterns of competence and adjustment among adolescents from authoritative, authoritarian, indulgent and neglectful families. Child Dev. 1991;62:1049–1065. 1756655 10.1111/j.1467-8624.1991.tb01588.x

[11] Simons LG, Conger RD. Linking mother-father differences in parenting to a typology of family parenting styles and adolescent outcomes. J Fam Issues. 2007;28(2):212–241.

[12] Wing Chan T, Koo A. Parenting style and youth outcomes in the UK. Eur Sociol Rev. 2011;27(3):385–399.

[13] German AJ. Style over substance: what can parenting styles tell us about ownership styles and obesity in companion animals? Br J Nutr. 2014;113:S72–S77. doi: 10.1017/S0007114514002335 25415870 10.1017/S0007114514002335

[14] Bray EE, Sammel MD, Cheney DL, Serpell JA, Seyfarth RM. Effects of maternal investment, temperament, and cognition on guide dog success. Proc Acad Nat Sci. 2017 8 7 doi: 10.1073/pnas.1704303114 28784785 10.1073/pnas.1704303114 PMC5576795

[15] Neidhart L, Boyd R. Companion Animal Adoption Study. J Appl Anim Welf Sci. 2002;5(3):175–192. doi: 10.1207/S15327604JAWS0503_02 12578739 10.1207/S15327604JAWS0503_02

[16] Kubinyi E, Turcsán B, Miklósi A. Dog and owner demographic characteristics and dog personality trait associations. Behav Processes. 2009;81(3):392–401. doi: 10.1016/j.beproc.2009.04.004 19520239 10.1016/j.beproc.2009.04.004

[17] Voith V. Attachment of people to companion animals. Vet Clin North Am Small Anim Pract. 1985;15:289–296. 3872510 10.1016/s0195-5616(85)50301-0

[18] Prato-Previde E, Custance DM, Spiezio C, Saratini F. Is the dog-human relationship an attachment bond? An observational study using Ainsworth’s strange situation. Behaviour. 2003;140:225–254.

[19] Archer J. Why do people love their pets? Evol Hum Behav. 1997;18:237–259.

[20] Nagasawa M, Kikusui T, Onaka T, Ohtta M. Dog's gaze at its owner increases owner's urinary oxytocin during social interaction. Horm Behav. 2009;55:434–441. doi: 10.1016/j.yhbeh.2008.12.002 19124024 10.1016/j.yhbeh.2008.12.002

[21] Jolliffe IT. Principal Components Analysis. New York: Springer-Verlag; 1986.

[22] Aunola K, Nurmi JE. The role of parenting styles in children's problem behavior. Child Dev. 2005;76(6):1144–1159. doi: 10.1111/j.1467-8624.2005.00841.x 16274431 10.1111/j.1467-8624.2005.00841.x

[23] Darling N, Steinberg L. Parenting style as context: An integrative model. Psychol Bull. 1993;113(3):487–496.

[24] Wood JJ, McLeod BD, Sigman M, Hwang WC, Chu BC. Parenting and childhood anxiety: Theory, empirical findings, and future directions. J Child Psychol Psychiatry. 2003;44(1):134–151. 12553416 10.1111/1469-7610.00106

[25] Beverland MB, Farrelly F, Ching Lim EA. Exploring the dark side of pet ownership: status- and control-based pet consumption. J Bus Res. 2008;61:490–496.

[26] Blouin DD. Are dogs children, companions or just animals? Understanding variations in people’s orientations towards animals. Anthrozoos. 2013;26(2):279–294.

[27] Larzelere RE, Sheffield Morris A, Harrist AW. Authoritative Parenting. Synthesizing Nurturance and Discipline for Optimal Child Development. 1st ed. Washington: American Psychological Association; 2013.

[28] Ascione FR, Weber CV, Thompson TM, Heath J, Maruyama M, Hayashi K. Battered pets and domestic violence: Animal abuse reported by women experiencing intimate violence and by non-abused women. Violence Against Women. 2007;13(4):354–373. doi: 10.1177/1077801207299201 17420515 10.1177/1077801207299201

[29] Becker F, French L. Making the links: child abuse, animal cruelty and domestic violence. Child Abuse Rev. 2004;13:399–414.

[30] Volant AM, Johnson JA, Gullone E, Coleman GJ. The relationship between domestic violence and animal abuse. J Interpers Violence. 2008;23(9):1277–1295. doi: 10.1177/0886260508314309 18326483 10.1177/0886260508314309

[31] Endenburg N, Knol BW. Behavioural, household, and social problems associated with companion animals: opinions of owners and non-owners. Vet Q. 1994;16:130–134. doi: 10.1080/01652176.1994.9694434 7985355 10.1080/01652176.1994.9694434

[32] Gazzano A, Maritia C, Sighieria C, Duccia M, Ciceronib C, McBride EA. Survey of undesirable behaviours displayed by potential guide dogs with puppy walkers. J of Vet Behav. 2008;3(3):104–113.

[33] Herron ME, Shofer FS, Reisner IR. Survey of the use and outcome of confrontational and non-confrontational training methods in client-owned dogs showing undesired behaviours. Appl Anim Behav Sci. 2009;117:47–54.

[34] Coleman GJ, Hemsworth PH, Hay M, Cox M. Modifying stockperson attitudes and behaviour towards pigs at a large commercial farm. Appl Anim Behav Sci. 2000;66:11–20.

[35] Hemsworth PH, Coleman GJ, Barnett JL. Improving the attitude and behaviour of stockpersons towards pigs and the consequences of the behaviour and reproductive performance of commercial pigs. Appl Anim Behav Sci. 1994;39:349–362.

[36] Hemsworth PH, Coleman GJ, Barnett JL, Borg S, Dowling S. The effects of cognitive behavioural intervention on the attitude and behaviour of stockpersons and the behaviour and productivity of commercial dairy cows. J Anim Sci. 2002;80:68–78. 11831530 10.2527/2002.80168x

