# Supplementary material for: The existence of parenting styles in the owner-dog relationship
Source: PLoS One. 2018 Feb 23;13(2):e0193471. doi: 10.1371/journal.pone.0193471 (PMC5825139; doi:10.1371/journal.pone.0193471)
Supplement: S1 Appendix — PSDQ items in the uninvolved style following Blakely Kimble (2009) and Baumrind (2013) (PDF) [file pone.0193471.s001.pdf]

S1 Appendix - Items measuring the uninvolved parenting style

PSDQ items in the uninvolved style following Blakely Kimble (2009) and Baumrind (2013)

	Parenting style	Element
Baumrind (2013) uninvolved style		
1. I encourage my child to talk about its troubles.	authoritative	warmth & involvement
3. I know the names of my child's friends.	authoritative	warmth & involvement
5. I give praise when my child is good.	authoritative	warmth & involvement
8. I withhold scolding and/or criticism even when my child acts contrary to my wishes.	permissive	ignoring misbehavior
9. I show sympathy when my child is hurt or frustrated.	authoritative	warmth & involvement
11. I spoil my child.[BK]	permissive	lack of follow through
12. I give comfort and understanding when my child is upset.	authoritative	warmth & involvement
15. I allow my child to annoy someone else.	permissive	ignoring misbehavior
20. I state punishments to my child and do not actually do them.[BK]	permissive	lack of follow through
21. I am responsive to my child's feelings or needs.	authoritative	warmth & involvement
27. I tell my child that we appreciate what it tries or accomplishes.	authoritative	warmth & involvement
33. I am aware of problems or concerns about my child in school.	authoritative	warmth & involvement
34. I threaten my child with punishment more often than actually giving it.[BK]	permissive	lack of follow through
35. I express affection by hugging, kissing, and holding my child.	authoritative	warmth & involvement
36. I ignore my child's misbehavior.	permissive	ignoring misbehavior
38. I carry out discipline after my child misbehaves.	permissive	lack of follow through
39. I apologize to my child when making a mistake in parenting	authoritative	warmth & involvement
41. I give into my child when it causes a commotion about something.[BK]	permissive	lack of follow through
45. I allow my child to interrupt others.	permissive	ignoring misbehavior
46. I have warm and intimate times together with my child.	authoritative	warmth & involvement
49. I promise rewards to my child to bring about compliance.	permissive	lack of follow through
Blakely Kimble (2009) uninvolved style		
4. I find it difficult to discipline my child.	permissive	self confidence
10. I punish by taking privileges away from my child with little if any explanations.	authoritarian	non-reasoning/punitive
11. I spoil my child.[B]	permissive	lack of follow through
13. I yell or shout when my child misbehaves.	authoritarian	verbal hostility
20. I state punishments to my child and do not actually do them.[B]	permissive	lack of follow through

28. I punish by putting my child off somewhere alone with little if any explanations.	authoritarian	non-reasoning/punitive
34. I threaten my child with punishment more often than actually giving it.[B]	permissive	lack of follow through
41. I give into my child when it causes a commotion about something.[B]	permissive	lack of follow through
54. I use threats as punishment with little or no justification.	authoritarian	non-reasoning/punitive
56. When my child asks why he/she has to conform, I state: because I said so, or I am your parent and I want you to.	authoritarian	non-reasoning/punitive
58. I explain the consequences of the child's behavior.	authoritative	reasoning/induction
62. I emphasize the reasons for rules.	authoritative	reasoning/induction

[B] – Item also in Baumrind-measurement of uninvolved style, [BK] – Item also in Blakely Kimble-measurement of uninvolved style
