# Supplementary material for: The existence of parenting styles in the owner-dog relationship
Source: PLoS One. 2018 Feb 23;13(2):e0193471. doi: 10.1371/journal.pone.0193471 (PMC5825139; doi:10.1371/journal.pone.0193471)
Supplement: S1 Table — Results of loadings after rotation from PCA-analysis for 32/62-PSDQ (Robinson et al., 1995) and new dog-directed parenting styles in 518 Dutch dog owning parents; presented are the loadings ≥ |0.4|. (PDF) [file pone.0193471.s002.pdf]

**S1 Table - Overview of PCA-item loadings**

Results of loadings after rotation from PCA-analysis for 32/62-PSDQ (Robinson et al., 1995) and new dog-directed parenting styles in 518 Dutch dog owning parents; presented are the loadings ≥ |0.4|.

| Item | μ±s.d. | Authoritarian 32-items | Authoritarian 62-items | Authoritarian - correction orientated (new DD-PSDQ) | Authoritative 32-items - intrinsic | Authoritative 62-items - intrinsic | Authoritative - intrinsic value orientated (new DD-PSDQ) | Authoritative 32-items - training | Authoritative 62-items - training | Authoritative - training orientated (new DD-PSDQ) |
|---|---|---|---|---|---|---|---|---|---|---|
| I use a poke of my finger, or short kick to snap my dog out of it when it misbehaves. [AN*] | 0.8±1.0 | -0.6 | 0.6 | 0.7 | - | - | - | - | - | - |
| I use short pulls on the leash or pull back when my dog pulls.[AN] | 1.6±1.2 | - | 0.6 | - | - | - | - | - | - | - |
| I allow my dog to jump up on people, as long as it is friendly. [PM] | 0.6±0.9 | - | - | - | - | - | - | - | - | - |
| I have good times together with my dog. [AV*] | 3.7±0.5 | - | - | - | - | - | - | - | - | - |
| When two dogs are fighting, I discipline first and think about why it happened later. [AN] | 2.1±1.4 | - | - | - | - | - | - | - | - | - |
| I encourage my dog to ‘be dog’ even when it results in a dirty or wet dog. [AV*] | 3.4±0.8 | - | - | - | - | - | - | - | 0.4 | - |
| I lure my dog with reward to solicit certain behaviour, even when it is misbehaving at that moment.[PM] | 2.4±1.3 | - | - | - | - | - | - | - | 0.5 | - |
| I scold or criticize when my dog's behaviour doesn't meet my expectations.[AN*] | 0.6±0.8 | -0.6 | 0.5 | 0.8 | - | - | - | - | - | - |
| I show respect for my dog' s needs by encouraging my dog to ‘be dog’.[AV*] | 3.4±0.8 | - | - | - | - | - | - | - | 0.4 | - |
| I set strict well-established rules for my dog.[PM] | 1.3±1.0 | - | - | - | - | - | - | - | - | - |
| I let my dog know how I feel about its good and bad behaviour.[AV*] | 2.9±1.0 | - | 0.4 | - | - | - | - | - | - | - |

| | | | | | | | | | | |
|---|---|---|---|---|---|---|---|---|---|---|
| I use threats as punishment without feeling need for justification towards my dog.AN* | 0.4±0.8 | -0.5 | 0.6 | - | - | - | - | - | - | - |
| I take into account my dog's preferences in making plans.AV* | 3.0±1.0 | - | - | - | 0.5 | 0.4 | 0.5 | - | - | - |
| When I ask my dog to do something, he should do so, because I said so and I am its boss.AN* | 2.2±1.2 | -0.5 | 0.6 | - | -0.5 | - | - | - | - | - |
| I am unsure on how to solve my dog's misbehaviour.PM | 0.9±1.0 | - | - | - | - | - | - | - | - | - |
| I practice behaviour step by step with my dog, so I am sure he understands what I ask of him.AV* | 3.1±1.0 | - | - | - | - | - | - | 0.6 | 0.6 | 0.6 |
| I demand that my dog does things.AN | 1.5±1.2 | - | 0.5 | - | - | - | - | - | - | - |
| I channel my dog's misbehaviour into a more acceptable activity.AV | 3.1±0.9 | - | - | - | - | - | - | - | 0.6 | - |
| I shove my dog when he is disobedient.AN | 0.7±0.9 | - | 0.6 | - | - | - | - | - | - | - |
| I use more or higher value reward (food or toy) when I believe my dog should really do something in a situation.AV* | 2.9±1.2 | - | - | - | - | - | - | 0.7 | 0.5 | 0.6 |
| I encourage my dog to show how it feels by its body language, I see growling as a signal of my dog’s emotion for example.AV* | 2.0±1.3 | - | - | - | 0.5 | 0.4 | 0.6 | - | - | - |
| I guide my dog by punishment more than by tapping into its natural needs.AN | 0.3±0.7 | - | 0.5 | - | - | - | - | - | - | - |
| I know the names of my dog’s play mates.AV | 3.1±1.1 | - | - | - | - | - | - | - | - | - |
| I find it difficult to discipline my dog.PM* | 0.8±1.0 | - | - | - | - | - | - | - | - | - |
| I give praise when my dog is good.AV* | 3.7±0.6 | - | - | - | - | - | - | 0.6 | 0.6 | 0.7 |

| | | | | | | | | | | |
|---|---|---|---|---|---|---|---|---|---|---|
| I use a corrective slap when my dog misbehaves.[AN*] | 0.7±1.0 | -0.7 | 0.7 | 0.8 | - | - | - | - | - | - |
| I play and have fun with my dog.[AV] | 3.7±0.6 | - | - | - | - | - | - | - | 0.5 | - |
| I set consequences when my dog acts contrary to my wishes.[PM] | 2.0±1.3 | - | -0.4 | - | - | - | - | - | -0.4 | - |
| I show sympathy when my dog is hurt or frustrated. [AV] | 3.0±1.1 | - | - | - | - | 0.6 | - | - | - | - |
| I punish by taking away toys from my dog.[AN*] | 0.2±0.7 | - | - | - | - | - | - | - | - | - |
| I spoil my dog.[PM*] | 2.3±1.1 | - | - | - | 0.6 | 0.6 | - | - | - | - |
| I give comfort when my dog is upset.[AV*] | 2.5±1.3 | - | - | - | 0.6 | 0.6 | 0.6 | - | - | - |
| I yell or shout when my dog misbehaves.[AN*] | 1.1±1.1 | -0.7 | 0.6 | 0.8 | - | - | - | - | - | - |
| I am easy going and relaxed with my dog.[AV] | 3.3±0.8 | - | - | - | - | - | - | - | - | - |
| I allow my dog to greet someone else, regardless of that person’s appreciation of dogs.[PM] | 1.2±1.2 | - | - | - | - | - | - | - | - | - |
| I practice certain behaviour with my dog before asking this behaviour in a more difficult situation.[AV] | 2.9±1.1 | - | - | - | - | - | - | - | 0.7 | - |
| I raise my voice to make my dog improve.[AN*] | 1.9±1.2 | -0.7 | 0.6 | 0.6 | - | - | - | - | - | - |
| I show patience with my dog.[AV] | 3.2±0.8 | - | - | - | - | - | - | - | - | - |
| I grab my dog when it is being disobedient.[AN*] | 1.1±1.1 | -0.6 | 0.6 | 0.5 | - | - | - | - | - | - |
| I threaten with punishments towards my dog and do not actually do them.[PM*] | 0.3±0.7 | -0.4 | 0.5 | - | - | - | - | - | - | - |
| I am responsive to my dog's feelings or needs.[AV*] | 3.2±0.9 | - | - | - | 0.5 | 0.4 | 0.6 | - | 0.5 | - |
| I allow my dog to give input on decisions for instance with regard to the route we follow on walks.[AV*] | 1.6±1.3 | - | - | - | 0.7 | 0.6 | 0.7 | - | - | - |
| I struggle with my dog.[AN] | 0.4±0.8 | - | - | - | - | - | - | - | - | - |

| | | | | | | | | | | |
|---|---|---|---|---|---|---|---|---|---|---|
| I am confident about training skills towards my dog.[PM] | 0.7±0.8 | - | - | - | - | - | - | - | - | - |
| I think about why rules should be obeyed by my dog.[AV*] | 3.3±0.9 | - | - | - | - | - | - | 0.6 | 0.6 | 0.6 |
| I am more concerned with own feelings than with my dog's feelings.[AN] | 1.1±1.0 | - | - | - | - | - | - | - | - | - |
| I tell my dog ‘good dog’ when he tries to follow guidance, even if he does not succeed.[AV] | 3.0±1.0 | - | - | - | - | - | - | - | - | - |
| I punish by giving my dog ‘time out’ and walking away if he misbehaves, even if he finds the situation he is in uncomforting.[AN*] | 1.1±1.2 | - | - | - | - | - | - | - | - | - |
| I help my dog to understand the impact of its behaviour by offering him choices in situations.[AV*] | 1.5±1.3 | - | - | - | - | - | - | - | - | - |
| I am afraid that disciplining my dog for misbehaviour will cause him to like me less.[PM] | 0.4±1.0 | - | - | - | - | - | - | - | - | - |
| I take my dog's desires into account before asking him to do something.[AV*] | 1.9±1.2 | - | - | - | 0.6 | 0.5 | 0.7 | - | - | - |
| I can explode in anger towards my dog when he does something he knows I don’t want him to do.[AN*] | 0.4±0.7 | -0.6 | 0.6 | 0.6 | - | - | - | - | - | - |
| I am aware of problems or concerns about my dog that neighbours (may) have.[AV] | 1.9±1.5 | - | - | - | - | - | - | - | - | - |
| I threaten my dog with punishment more often than actually giving it.[PM*] | 0.4±0.8 | - | - | - | - | - | - | - | - | - |
| I express affection, for instance by rubbing my dog under its chin.[AV] | 3.6±0.7 | - | - | - | - | - | - | - | - | - |
| I ignore my dog's misbehaviour such as chasing game, barking | 0.9±1.2 | - | - | - | - | - | - | - | - | - |

| | | | | | | | | | | |
|---|---|---|---|---|---|---|---|---|---|---|
| at other people or peeing against stores in a shopping area.[PM] | | | | | | | | | | |
| I use physical punishment as a way to improve my dog's behaviour.**[AN*]** | 0.6±1.0 | -0.6 | 0.6 | 0.8 | - | - | - | - | - | - |
| I carry out discipline after my dog misbehaves.**[PM]** | 1.7±1.2 | - | - | - | - | - | - | - | - | - |
| I feel bad towards my dog when making a mistake in guiding it.**[AV]** | 1.9±1.3 | - | - | - | - | - | - | - | - | - |
| I let my dog know what I expect from him.**[AV]** | 3.2±0.8 | - | - | - | - | - | - | - | - | - |
| I give into my dog when he causes a commotion about something or doesn't do something I want it to.[PM*] | 0.8±1.0 | - | - | - | - | 0.5 | - | - | - | - |
| I think about why my dog does something when it misbehaves.[AV] | 3.2±0.9 | - | - | - | - | - | - | - | 0.6 | - |

[AN] - Authoritarian item in the original PSDQ, [AV] - Authoritative item in the original PSDQ, [PM] - Permissive item in the original PSDQ, [*] - 32-PSDQ
