# Supplementary material for: The existence of parenting styles in the owner-dog relationship
Source: PLoS One. 2018 Feb 23;13(2):e0193471. doi: 10.1371/journal.pone.0193471 (PMC5825139; doi:10.1371/journal.pone.0193471)
Supplement: S2 Table — Dutch dog owning parents (N = 518) reported on child-directed parenting in 32 items adapted from the Parenting Styles and Dimensions Questionnaire (PSDQ). Answers on a five-point Likert scale were analysed by Principal Component Analysis and presented are the loadings ≥ |0.4| and percentages of variation explained by the main components, which represented dimensions of parenting authoritatively, authoritarian and permissively. (PDF) [file pone.0193471.s003.pdf]

S2 Table - 32-item child-directed PSDQ Principal Component Analysis

Dutch dog owning parents (*N*=518) reported on child-directed parenting in 32 items adapted from the Parenting Styles and Dimensions Questionnaire (PSDQ). Answers on a five-point Likert scale were analysed by Principal Component Analysis and presented are the loadings ≥ |0.4| and percentages of variation explained by the main components, which represented dimensions of parenting authoritatively, authoritarian and permissively.

**Item**	**Variance explained (latent root)**		
	23% (7.4) Authoritative	15% (4.9) Authoritarian	6% (1.9) Permissive
I emphasize the reasons for rules.[AV]	0.5		
I encourage my child to talk about the child's troubles.[AV]	0.7		
I give praise when my child is good.[AV]	0.7		
I give comfort and understanding when my child is upset.[AV]	0.8		
I am responsive to my child's feelings or needs.[AV]	0.7		
I give my child reasons why rules should be obeyed.[AV]	0.7		
I help my child to understand the impact of behaviour by encouraging my child to talk about the consequences of his/her own actions.[AV]	0.6		
I have warm and intimate times together with my child.[AV]	0.7		
I show respect for my child' s opinions by encouraging my child to express them.[AV]	0.7		
I explain to my child how I feel about the child's good and bad behaviour.[AV]	0.7		
I explain the consequences of the child's behaviour.[AV]	0.8		
I spank when my child is disobedient.[AN]		0.8	
I punish by taking privileges away from my child with little if any explanations.[AN]		0.4	
I grab my child when he/she is being disobedient.[AN]		0.6	
I punish by putting my child off somewhere alone with little if any explanations [AN]		0.5	
I use physical punishment as a way of disciplining my child.[AN]		0.6	
I slap my child when the child misbehaves.[AN]		0.9	
I use threats as punishment with little or no justification.[AN]		0.4	
I find it difficult to discipline my child.[PM]			-0.4
I spoil my child.[PM]			-0.5
I state punishment to my child and do not actually do them.[PM]			-0.8
I give into my child when the child causes a commotion about something.[PM]			-0.4
I use threats as punishment with little or no justification.[AN!]			-0.4

[AN] - Authoritarian item in the original PSDQ, [AV] - Authoritative item, [PM] - Permissive item

[!] - Item scoring in a different PSDQ dimension than found originally by Robinson et al. (1995)
