# Supplementary material for: The existence of parenting styles in the owner-dog relationship
Source: PLoS One. 2018 Feb 23;13(2):e0193471. doi: 10.1371/journal.pone.0193471 (PMC5825139; doi:10.1371/journal.pone.0193471)
Supplement: S3 Table — Dutch dog owning parents (N = 518) reported on dog-directed parenting in 62 items adapted from the Parenting Styles and Dimensions Questionnaire (PSDQ). Answers on a five-point Likert scale were analysed by Principal Component Analysis and presented are the loadings ≥ |0.4| and percentages of variation explained by the main components, which represented dimensions of parenting authoritatively and authoritarian. (PDF) [file pone.0193471.s004.pdf]

S3 Table - 62-item dog-directed PSDQ Principal Component Analysis

Dutch dog owning parents (*N*=518) reported on dog-directed parenting in 62 items adapted from the Parenting Styles and Dimensions Questionnaire (PSDQ). Answers on a five-point Likert scale were analysed by Principal Component Analysis and presented are the loadings ≥ |0.4| and percentages of variation explained by the main components, which represented dimensions of parenting authoritatively and authoritarian.

Item	Variance explained (latent root)		
	13% (8.0) Authoritative	9% (5.3) Authoritarian	7% (4.5) Authoritative - intrinsic value/emotion
I practice certain behaviour with my dog before asking this behaviour in a more difficult situation.[AV]	0.7		
I practice behaviour step by step with my dog, so I am sure he understands what I ask of him.[AV]	0.6		
I think about why my dog does something when it misbehaves.[AV]	0.6		
I channel my dog's misbehaviour into a more acceptable activity.[AV]	0.6		
I think about why rules should be obeyed by my dog.[AV]	0.6		
I give praise when my dog is good.[AV]	0.6		
I use more or higher value reward (food or toy) when I believe my dog should really do something in a situation.[AV]	0.5		
I play and have fun with my dog.[AV]	0.5		
I lure my dog with reward to solicit certain behaviour, even when it is misbehaving at that moment.[PM!]	0.5		
I am responsive to my dog's feelings or needs.[AV]	0.5		
I encourage my dog to ‘be dog’ even when it results in a dirty or wet dog.[AV]	0.4		
I show respect for my dog' s needs by encouraging my dog to ‘be dog’.[AV]	0.4		
I set consequences when my dog acts contrary to my wishes.[PM!]	-0.4		
I use a corrective slap when my dog misbehaves.[AN]		0.7	
I use physical punishment (for instance a slap or a correction chain) as a way to improve my dog’s behaviour.[AN]		0.6	
I use short pulls on the leash or pull back when my dog pulls.[AN]		0.6	
I grab my dog when he is being disobedient.[AN]		0.6	
I raise my voice to make my dog improve.[AN]		0.6	

I yell or shout when my dog misbehaves.[AN]	0.6	
When I ask my dog to do something, he should do so, because I said so and I am its boss.[AN]	0.6	
I shove my dog when he is disobedient.[AN]	0.6	
I use threats as punishment without feeling need for justification towards my dog.[AN]	0.6	
I can explode in anger towards my dog when he does something he knows I don't want him to do.[AN]	0.6	
I use a poke of my finger, or short kick to snap my dog out of it when it misbehaves.[AN]	0.6	
I demand that my dog does things.[AN]	0.5	
I guide my dog by punishment more than by tapping into its natural needs.[AN]	0.5	
I scold or criticize when my dog's behaviour doesn't meet my expectations.[AN]	0.5	
I threaten with punishments towards my dog and do not actually do them.[PM!]	0.5	
I let my dog know how I feel about its good and bad behaviour.[AV!]	0.4	
I set consequences when my dog acts contrary to my wishes.[PM!]	-0.4	
I give comfort when my dog is upset.[AV]		0.6
I show sympathy when my dog is hurt or frustrated.[AV]		0.6
I spoil my dog. [PM!]		0.6
I allow my dog to give input on decisions for instance with regard to the route we follow on walks.[AV]		0.6
I give into my dog when he causes a commotion about something or doesn't do something I want it to.[PM!]		0.5
I take my dog's desires into account before asking him to do something.[AV]		0.5
I am responsive to my dog's feelings or needs.[AV]		0.4
I encourage my dog to show how it feels, it is allowed to growl for instance, when uncomfortable.[AV]		0.4
I take into account my dog's preferences in making plans.[AV]		0.4

[AN] - Authoritarian item in the original PSDQ, [AV] - Authoritative item, [PM] – Permissive item
[*] - Item in 32-PSDQ
