# Supplementary material for: The existence of parenting styles in the owner-dog relationship
Source: PLoS One. 2018 Feb 23;13(2):e0193471. doi: 10.1371/journal.pone.0193471 (PMC5825139; doi:10.1371/journal.pone.0193471)
Supplement: S4 Table — Dutch dog owners (N = 518) filled out a 32-item Parenting Styles and Dimensions Questionnaire (PSDQ) adapted for assessing dog-directed parenting styles. Answers on a five-point Likert scale were analysed by Principal Components Analysis, omitting items with loadings < │0.4│for the main components. Presented are the final outcomes on eighteen items with loadings ≥ |0.4| and percentages of variation explained by two components of parenting authoritarian-correction orientated (two times four items), one component of authoritative-intrinsic value orientated (six items) and one of authoritative-training orientated (four items), together explaining 48% of variation. (PDF) [file pone.0193471.s005.pdf]

S4 Table - Second step 32-item dog-directed PSDQ Principal Component Analysis

Dutch dog owners (*N*=518) filled out a 32-item Parenting Styles and Dimensions Questionnaire (PSDQ) adapted for assessing dog-directed parenting styles. Answers on a five-point Likert scale were analysed by Principal Components Analysis, omitting items with loadings < |0.4| for the main components. Presented are the final outcomes on eighteen items with loadings ≥ |0.4| and percentages of variation explained by two components of parenting authoritarian-correction orientated (two times four items), one component of authoritative-intrinsic value orientated (six items) and one of authoritative-training orientated (four items), together explaining 48% of variation.

	**Variation explained (latent root)**			
	22% (4.6)	6% (1.2)	12% (2.6)	8% (1.8)
	Authoritarian-correction orientated-verbal	Authoritarian-correction orientated-physical	Authoritative-intrinsic value orientated	Authoritative-training orientated
Authoritarian – correction orientated - verbal				
I yell or shout when my dog misbehaves.[AN]	0.76			
I scold or criticize when my dog's behaviour doesn't meet my expectations.[AN]	0.75			
I can explode in anger towards my dog when he does something he knows I don't want him to do.[AN]	0.61			
I raise my voice to make my dog improve.[AN]	0.55			
Authoritarian – correction orientated - physical				
I use physical punishment (for instance a slap or a correction chain) as a way to improve my dog's behaviour.[AN]		0.79		
I use a corrective slap when my dog misbehaves.[AN]		0.75		
I use a poke of my finger, or short kick to snap my dog out of it when it misbehaves.[AN]		0.74		
I grab my dog when he/she is being disobedient.[AN]		0.45		
Authoritative – intrinsic value orientated				
I allow my dog to give input on decisions for instance with regard to the route we follow on walks.[AV]			0.73	
I take my dog's desires into account before asking him to do something.[AV]			0.65	
I am responsive to my dog's feelings or needs.[AV]			0.61	
I encourage my dog to show how it feels, it is allowed to growl for instance, when uncomfortable.[AV]			0.58	
I give comfort when my dog is upset.[AV]			0.55	
I take into account my dog's preferences in making plans.[AV]			0.51	
Authoritative – training orientated				
I give praise when my dog is good.[AV]				0.69
I practice behaviour step by step with my dog, so I am sure he understands what I ask of him.[AV]				0.64
I use more or higher value reward (food or toy) when I believe my dog should really do something in a situation.[AV]				0.58
I think about why rules should be obeyed by my dog.[AV]				0.58

[AN] - Authoritarian item in the original PSDQ, [AV] - Authoritative item in the original PSDQ
